# Natural Dissociation Ratio of Carboxyl Group Controlled Highly Dispersed Silver Nanoparticles on PSA Microspheres and Their Catalytic Performance

**DOI:** 10.1186/s11671-018-2824-7

**Published:** 2018-12-18

**Authors:** Xiaoyu Zhao, Yingbing Zhang, Jin Zhang, Peijie Xue, Yanfei Wang, Rui Liu, Ruge Cao, Liang Zhu, Gang Li, Zuoliang Sha

**Affiliations:** 1 Tianjin Key Laboratory of Marine Resources and Chemistry, College of Chemical Engineering and Materials Science, Tianjin University of Science and Technology, Tianjin, 300457 China; 2 State Key Laboratory of Environmental Chemistry and Ecotoxicology, Research Center for Eco-Environmental Sciences, Chinese Academy of Sciences, Beijing, 100085 China; 3 College of Food Engineering and Biotechnology, Tianjin University of Science and Technology, Tianjin, 300457 China

**Keywords:** Poly(styrene-co-acrylic acid) microspheres, Silver nanoparticles, Distance-variable parallel electrodes system, Carboxyl dissociation ratio, Catalytic performance

## Abstract

The highly dispersed silver nanoparticle-loaded poly(styrene-co-acrylic acid) nanocomposites (nAg@PSA) were prepared and characterized by transmission electron microscopy and thermogravimetry. The amount and distribution of colloidal silver per particle were related to the dissociation ratio of carboxyl groups in the PSA sphere. The amount of carboxyl groups was evaluated by a conductivity titration curve. However, the dissociation of carboxyl groups on PSA is difficult to determine accurately via existing methods because the dissociation ratio will increase with increasing impurity ions during titration. We developed a technique to determine the dissociation ratio of PSA without impurity ions. This employs a novel distance-variable parallel electrode system. Thus, the relationship between nano silver distribution and natural dissociation of carboxyl groups on the surface of the PSA spheres was investigated for the first time. Accurately measuring and controlling the dissociation facilitated the production of PSA spheres containing highly dispersed silver nanoparticles. The catalytic performance of as-prepared nAg@PSA catalysts was studied by reduction of 4-nitrophenol. By controlling the amount of natural dissociation ratio of carboxyl group on PSA sphere, dispersion of silver nanoparticles can be designed and attained controllably. They offer easy synthesis, high catalytic performance, and good recyclability.

## Introduction

Recently, the preparation of core-shell composite microspheres comprising a dielectric solid sphere covering a metallic shell has attracted much attention. This interest was triggered by their unique catalytic and optical properties. These core-shell microspheres have substantial potential across a wide range of applications, such as surface-enhanced Raman scattering (SERS) [1–5], catalysis [6–10], nanoengineering of optical resonances [11, 12], photonic crystals [13–15], or biochemistry [16, 17] for such applications as chemical sensors. Many efforts have been focused on preparing core-shell composite nanospheres with noble metallic shells due to their novel optical and catalytic properties [18]. One of the most frequently studied systems is composite microspheres with silver shells. Various synthetic methods have been studied, including self-assembly [19], seeding-plating [18], successive ion layer adsorption and reaction (SILAR) [20], and in situ reduction deposition [21].

However, there are few reports on the controlled preparation of silver shells on poly(styrene-*co*-acrylic acid) microsphere surfaces. The amount of silver nanoparticles supported on poly(styrene-*co*-acrylic acid) composite nanospheres is determined by several factors, including the temperature, the amount of carboxyl groups, and the amount of the dissociated charges around the PSA spheres. The effect of temperature on the deposition of Ag nanoparticles has been explored and described in the literature [22]. The amount of carboxyl groups has been studied with conductivity titration methods [19]. The number of dissociated charges is generally smaller than the stoichiometric number because the behavior of the weak acid depends on the ionic strength. Adding electrolyte salts during electrochemical measurements immeasurably increases the dissociation ratio of the carboxyl groups. Consequently, quantitatively evaluating the amount of the dissociated charges in the latex suspensions is difficult. Nevertheless, the amount of dissociated carboxyl groups per particle affects the deposition of silver nanoparticles and was studied here by taking advantage of a distance-variable parallel electrode system. This facilitates the controllable preparation of silver nanoparticles.

Some applications of silver nanoparticle-loaded poly(styrene-*co*-acrylic acid) nanospheres have been reported [22–24]. Li and co-workers [22] prepared silver nanoparticle-coated poly(styrene-*co*-acrylic acid) composite nanospheres and then used these nanospheres as surface-enhanced Raman spectroscopy (SERS) substrates. Song and co-workers [24] synthesized silver nanoparticle-loaded poly(styrene-*co*-acrylic acid) nanospheres as antibacterial agents. However, little research has been done on the catalytic applications of silver nanoparticle-loaded poly(styrene-*co*-acrylic acid). Surprisingly, there is no report on the relationship between the dispersion of the Ag nanoparticles and the dissociation ratio of carboxyl groups. Our previous work [23] has reported a rough relationship between carboxyl groups and nano silver particles by thermogravimetry and TEM results. This time we raise a new viewpoint based on natural dissociation ratio of carboxyl group by developing a novel accurate electrochemical measurement.

This report describes the accurate characterization of natural dissociation ratio of carboxyl group of PSA spheres via two wire electrodes to prevent entry of impurities. We synthesized four sizes of latex particles to vary the value of the dissociated charges. The formation of well-distributed silver nanoparticles located on PSA nanospheres then was investigated. Poly(styrene-*co*-acrylic acid) nanospheres offer a large surface area for immobilization of silver nanoparticles and controllable dissociated carboxyl amount for good distribution of silver nanoparticles. This part seems similar with our previously work [23]. However, there is one critical progress in this work that needs to be pointed out: It is natural dissociation ratio of carboxyl group that fundamentally decide the numbers of Ag nanoparticles rather than the total numbers of carboxyl group. This conclusion only can be established on accurate measurement of natural dissociation ratio of carboxyl group. Such highly dispersed silver nanoparticles on the nAg@PSA nanocomposite show high catalytic performance by using the reduction of 4-nitrophenol as a model reaction. This has interesting potential for future studies.

## Results and Discussion

### Amount of Carboxyl Groups in the PSA Nanospheres

**Fig. 1:**
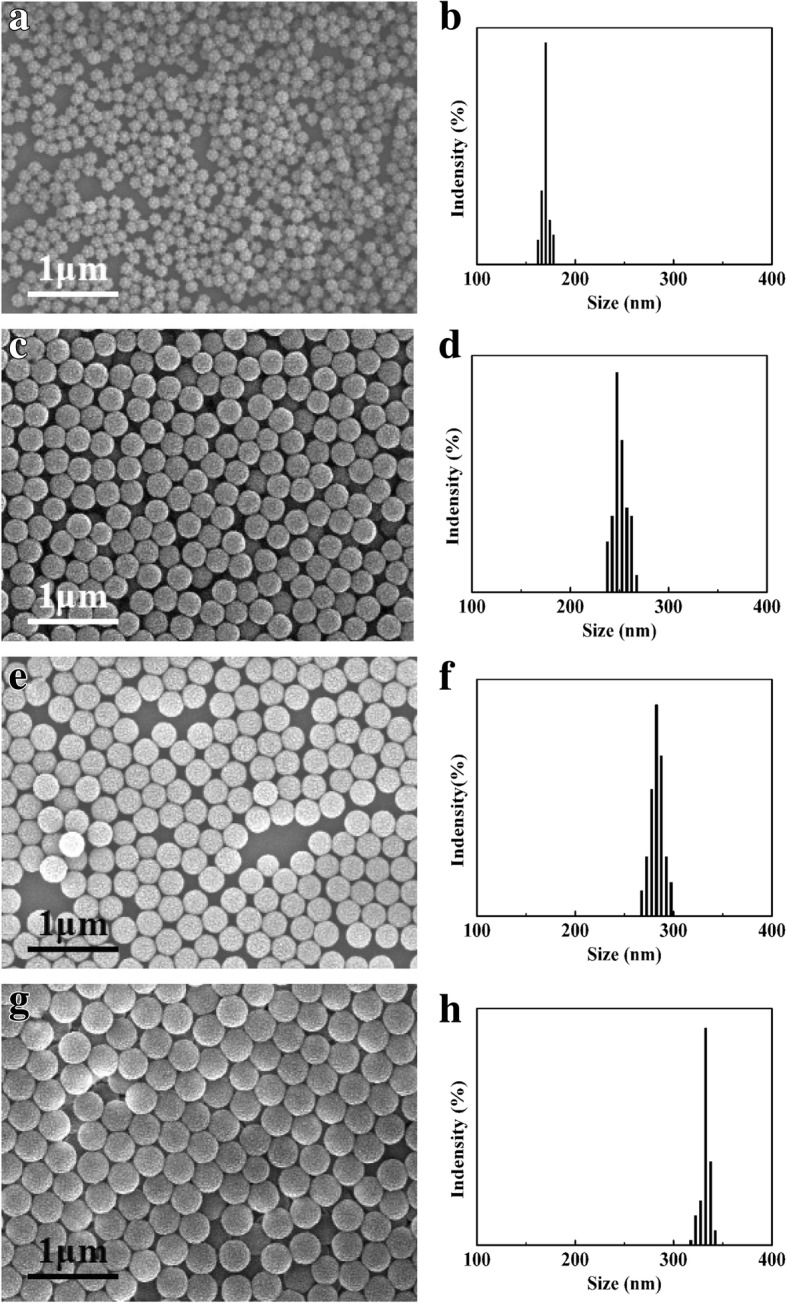
SEM micrographs and the particle size distribution histograms of PSA nanospheres: **a**, **b** PSA1; **c**, **d** PSA2; **e**, **f** PSA3; **g**, **h** PSA4

Figure 1 shows SEM micrographs and the corresponding particle size distributions of four kinds of PSA nanospheres. The particles were spherical with well-controlled particle size distributions. The particle sizes were studied via SEM, and the volume of the nanospheres was calculated. The sizes are listed in Table 1. The nanosphere volume, suspension density of PSA suspension, and number of spheres in the stock suspensions were determined by a previously published method [23, 25–27].

**Table 1:** Properties of four PSA suspensions

Sample code	2*r*0 ^(1)^ (nm)	*n* ^(2)^	*c* ^(3)^ (mol L^−1^)	*Λ* ^(4)^	*z* ^(5)^ (S m2 mol^−1^)
PSA1	169.65 ± 3.56	3.84 × 10^5^	1.88 × 10^−8^	3.42 × 10^2^	4.23 × 10^3^
PSA2	250.91 ± 7.36	7.20 × 10^5^	4.44 × 10^−9^	8.61 × 10^2^	8.71 × 10^3^
PSA3	283.16 ± 7.39	1.13 × 10^6^	1.94 × 10^−9^	1.63 × 10^3^	1.34 × 10^4^
PSA4	332.10 ± 4.80	1.60 × 10^6^	6.77 × 10^−9^	2.30 × 10^3^	1.74 × 10^4^

^(1)^*r*_0_, the radii of PSA nanospheres

^(2)^*n*, the number of the −COOH per particle

^(3)^*c*, molar concentration of PSA nanospheres

^(4)^*Λ*, the molar conductivity of the PSA suspensions

^(5)^*z*, the number of −COO^+^ per particle

**Fig. 2:**
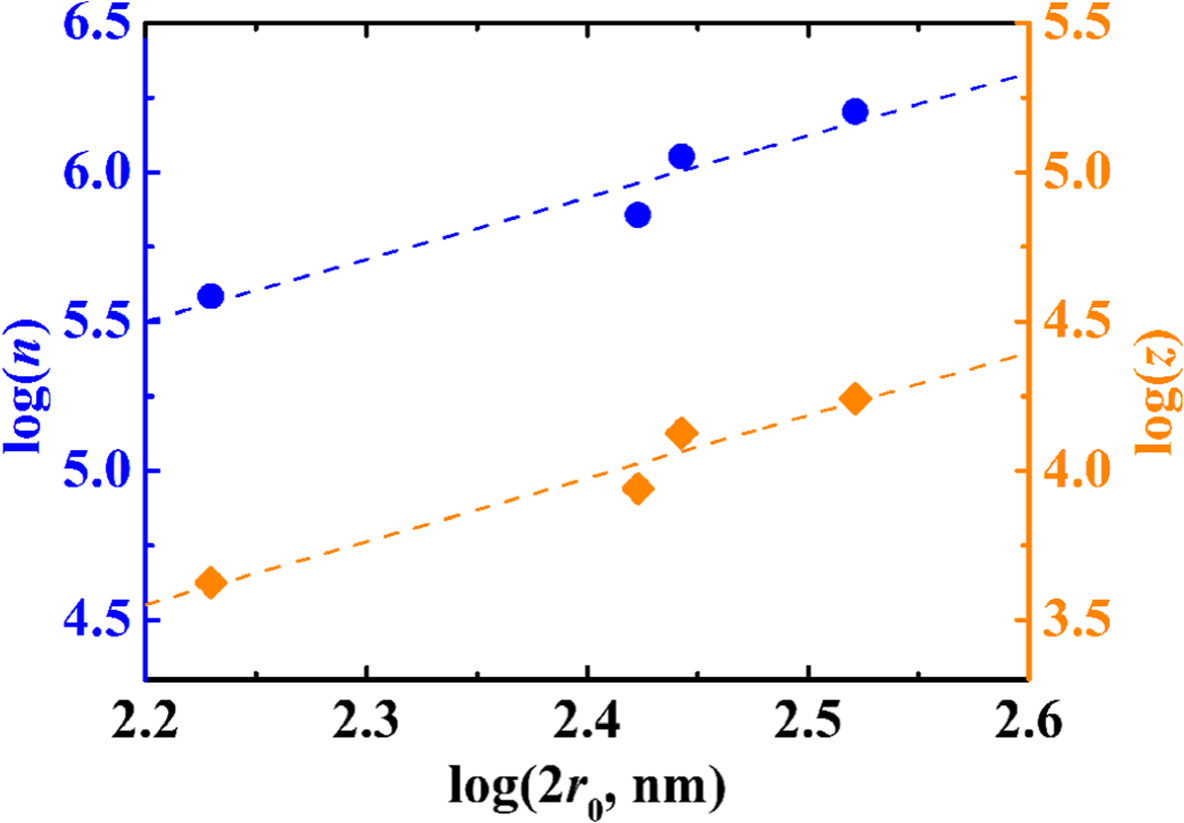
Logarithmic plots of the number of −COOH and −COO^+^ per particle against the diameters of PSA spheres

The amount of carboxyl groups on the particle was determined by conductivity titration which was reported before [23]. The carboxyl loading levels (per particle) are listed in Table 1. Figure 2 shows a logarithmic plot of the total numbers of carboxyl group per particle with the diameters of PSA sphere. The plot shows a linear relation with a slope of 2.0. This is an evidence that the carboxyl group may mainly be distributed in the spherical surface rather than phase volume.

### Natural Dissociation Ratio of Carboxyl Groups on PSA Nanospheres

The cell was filled with a PSA suspension of known latex concentration. Then, AC voltage of 10 mV was applied to obtain the AC impedance of suspension between parallel wire electrodes. The expression for the solution resistance between two parallel wires is approximately [22]

1 $$ {R}_S=0.916\frac{\log \left(d/a-1\right)}{Lc\varLambda} $$

**Fig. 3:**
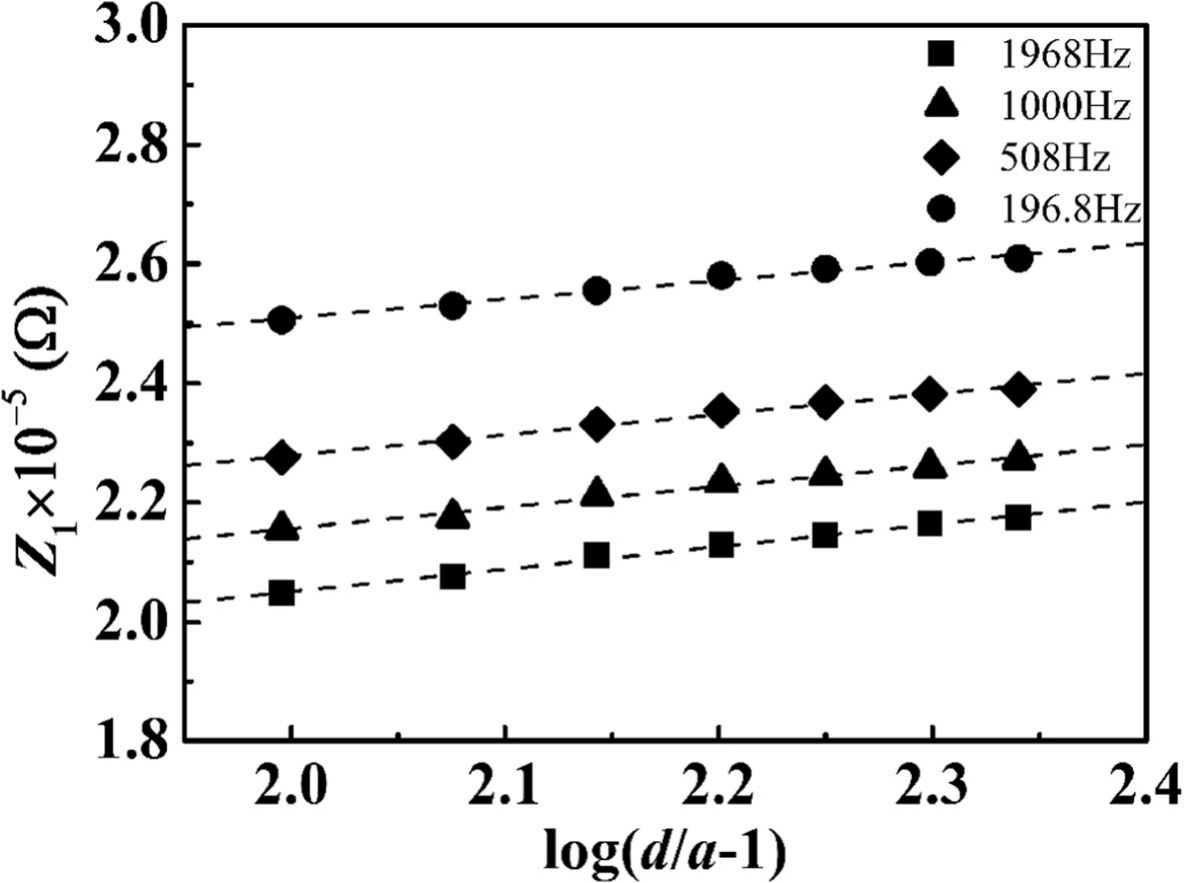
Plot of the real part of the AC impedance of a typical PSA suspension against log(*d*/*a* − 1) for frequencies *f* = 196.8, 508, 1000, and 1968 Hz, respectively

Here, *Λ* is the molar conductivity of the PSA suspension with concentration *c*, *d* is the distance between two electrodes, *a* is the radius of Pt wire, and *L* is the length of Pt wire immersed in suspension. Resistance of the suspension was obtained by Nyquist plots. Values of *Z*_1_ were plotted against log(*d*/*a* − 1) for some frequencies in Fig. 3. The plot for a given frequency fell on a line with the common slope. This exhibits positive intercepts at log(*d*/*a* − 1) = 0 or *d* = 2*a*. The linear plot was partially supported by Eq. (1), and it did not satisfy the proportionality of Eq. (1) in the appearance (positive values of the intercepts). The intercept means that the resistance would appear if the two electrodes were to come in contact to each other. This resistance should be located at the interface or included in the double layer. The linearity slope that equals to 0.916/*LcΛ*, according to Eq. (1), should be independent of frequency. The molar conductivity values (based on molar concentration of the PSA spheres, by regarding a PSA sphere as a huge charge carrier) were calculated and averaged over frequencies more than 150 Hz from the slope; these are listed in Table 1. Since one PSA sphere carries a huge number of −COOH, those values are much larger than ordinary ions. Figure 3 shows the dependence of the molar conductivity on the diameters of PSA sphere. They lie on a straight line with a slope of 2.9 suggesting that $\varLambda ={kr}_0^3$. This implies that the large particles should extremely enhance the molar conductivity since the accumulation of charges is on a single sphere.

As we described before [23, 26], the molar conductivity of the latex suspension per *N*_A_ latex particles were defined as the sum of the molar conductivity of *z* free hydrogen ions, *zλ*_H_, and the conductivity of *N*_A_ left *z* charged −COO^—^ carrier PSA sphere, *λ*_L_:

2 $$ {\varLambda}_{\mathrm{L}}=z{\lambda}_{\mathrm{H}}+{\lambda}_{\mathrm{L}} $$

Ionic conductivity is represented in terms of the diffusion coefficient *D*, through

3 $$ \lambda =\frac{Dz^2{F}^2}{RT} $$

When Eq. (3) for hydrogen ion, *λ*_H_ = *F*^2^*D*_H_/*RT*, and the latex particle, *λ*_L_ = *F*^2^*z*^2^*D*_L_/*RT*are inserted into Eq. (2), we obtain

4 $$ {\varLambda}_{\mathrm{L}}=\left({F}^2/ RT\right)\left({zD}_{\mathrm{H}}+{z}^2{D}_{\mathrm{L}}\right) $$

**Fig. 4:**
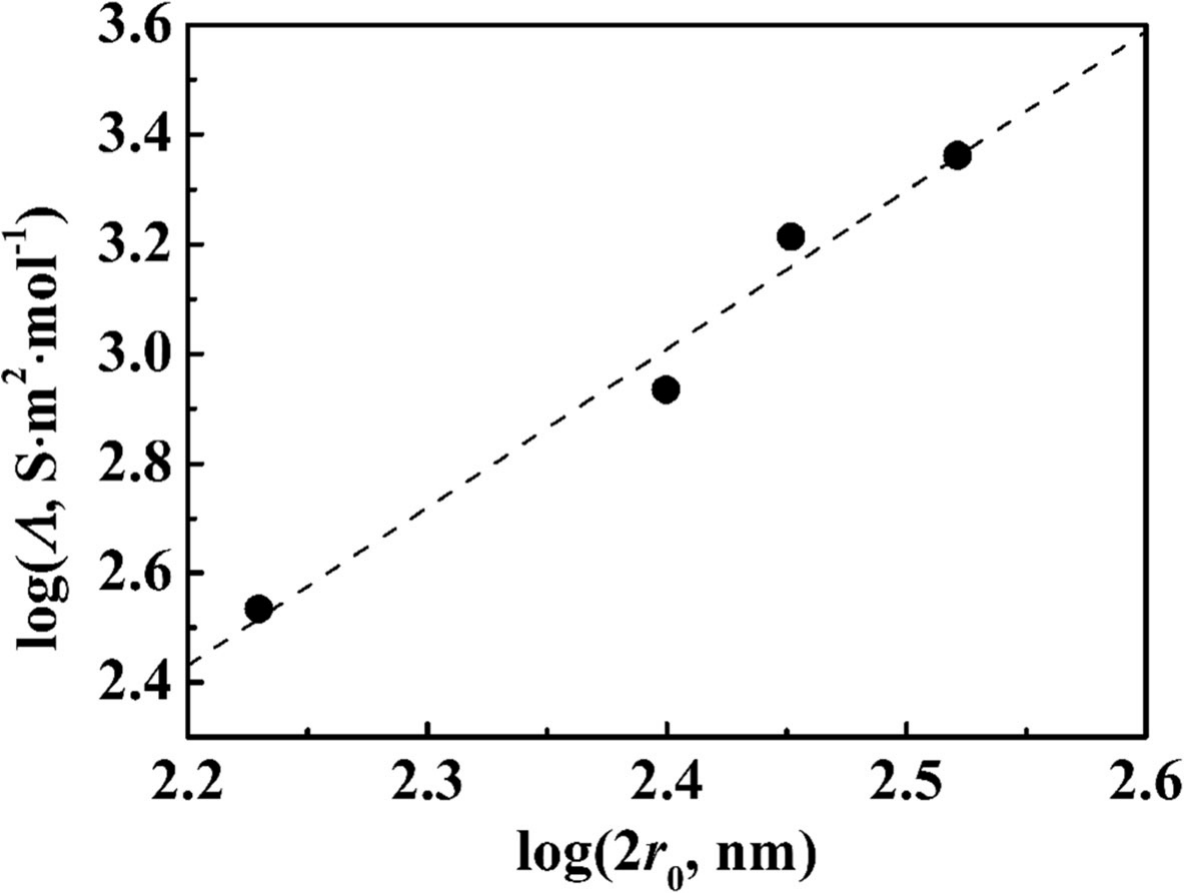
Variation of the molar conductivity with the diameters for PSA suspensions in logarithmic scale

Here, *D*_H_ and *D*_L_ represent the diffusion coefficients of hydrogen ion and −COO^—^ carrier PSA sphere, respectively. *D*_*L*_ was estimated by the Stokes–Einstein equation, and then inserted to Eq. (4) with *D*_H_ (9.3 × 10^−9^ m^2^ s^−1^), solving the equation for *z*. The results are listed in Table 1. Figure 4 shows the plot of *z* with diameters of a PSA sphere. The slope of the line is 2.1, which is nearly equal to the slope of the linearity of *n* against 2*r*_0_. The value of *z*/*n* was 0.01. This was calculated according to Table 1, which represents the dissociation of the carboxyl in a particle as

5A $$ {\left(-\mathrm{COOH}\right)}_n\leftrightarrow {\left(-\mathrm{COOH}\right)}_{n-z}\ {\left(-{\mathrm{COO}}^{-}\right)}_z+z{\mathrm{H}}^{+}\ \left(100\leftrightarrow 99:1:1\right) $$

This value indicates that the natural dissociation ratio of –COOH was only 1%. The other portion is in the neutral form of –COOH.

By inserting this result in Eq. (5A):

5B $$ {\left(-\mathrm{COOH}\right)}_{100}\leftrightarrow {\left(-\mathrm{COOH}\right)}_{99}{\mathrm{COO}}^{-}+{\mathrm{H}}^{+}. $$

The conductance is mainly caused by the formation of (AH)_99_A^−^ and H^+^ through the reaction (5B).

**Fig. 5:**
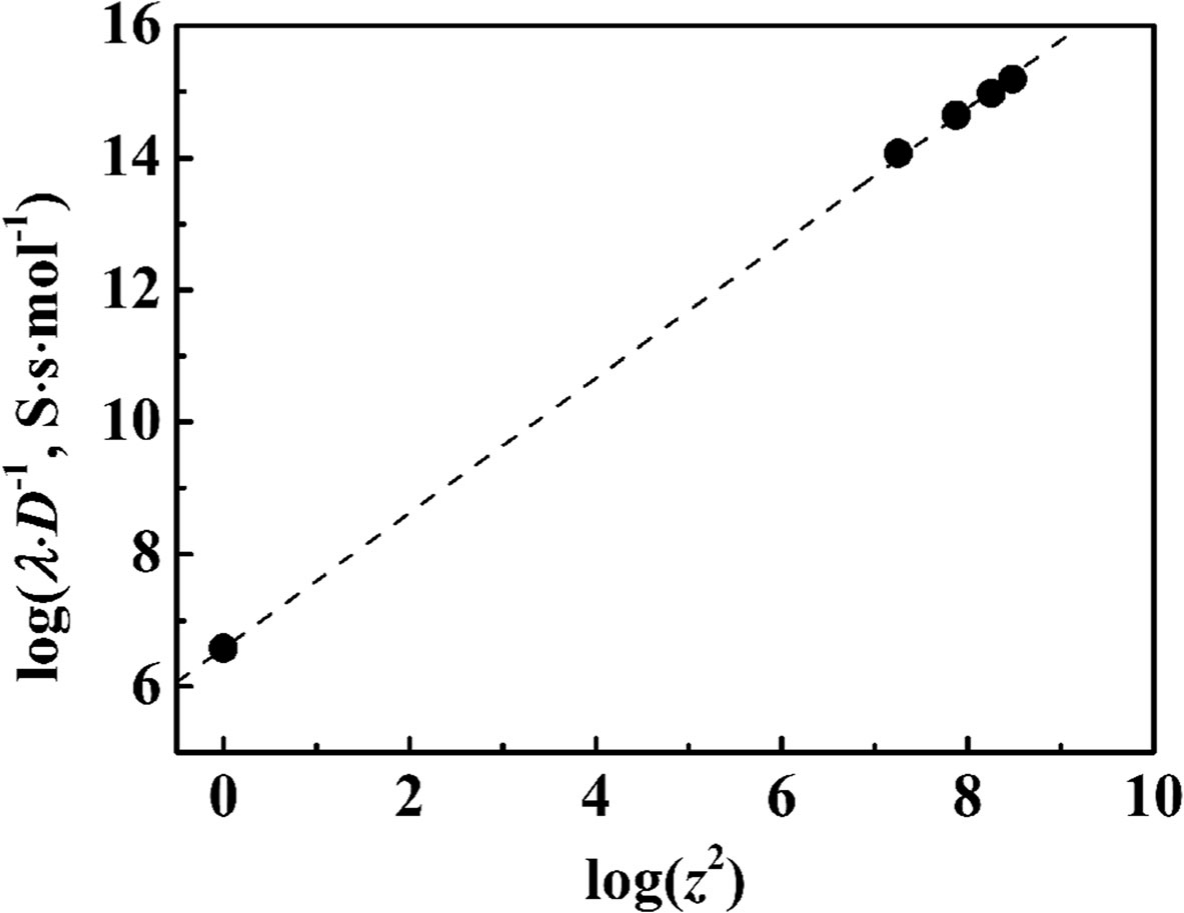
Logarithmic plot of *λ*·*D*^−1^ against *z*^2^ for the four PSA suspensions and halide ions

In order to confirm the reliability of this methods and as obtained results, we plotted the calculated values of *λ*_L_/*D* against *z*^2^ for the four latex particles and monovalent materials (for *z* = 1) on a logarithmic scale in Fig. 5. As expected, it shows that a straight line passed through values of monovalent materials. According to Eq. (3), the values of *λ*/*Dz*^2^ (= *F*^2^/*RT*) should be constant and independent of the diameters or other properties of the particles.

### Mechanism of Silver Nanoparticles Supported on PSA Nanospheres

**Fig. 6:**
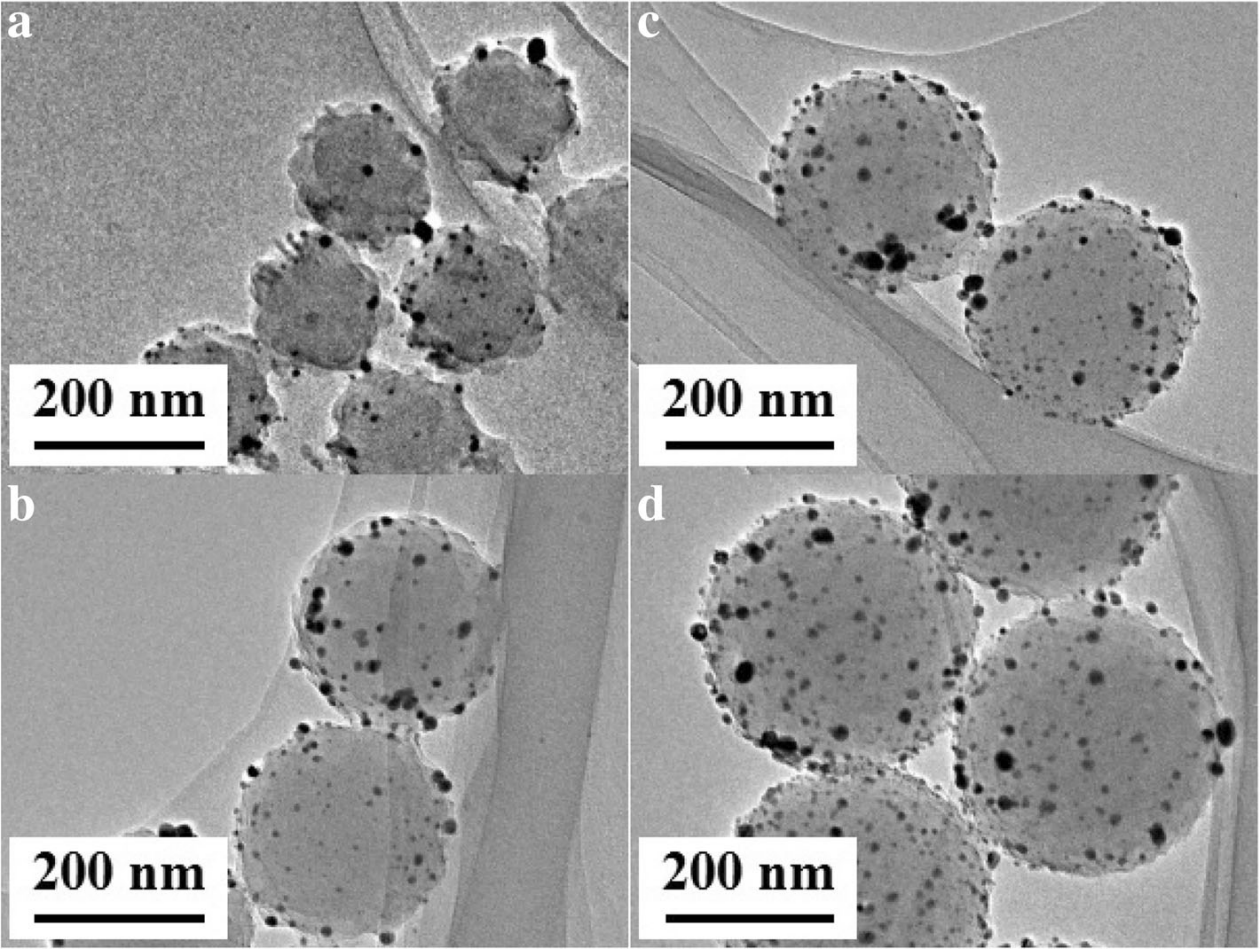
TEM images of nAg@PSA nanocomposites. **a**–**d**, corresponding to nAg@PSA1–nAg@PSA4

**Fig. 7:**
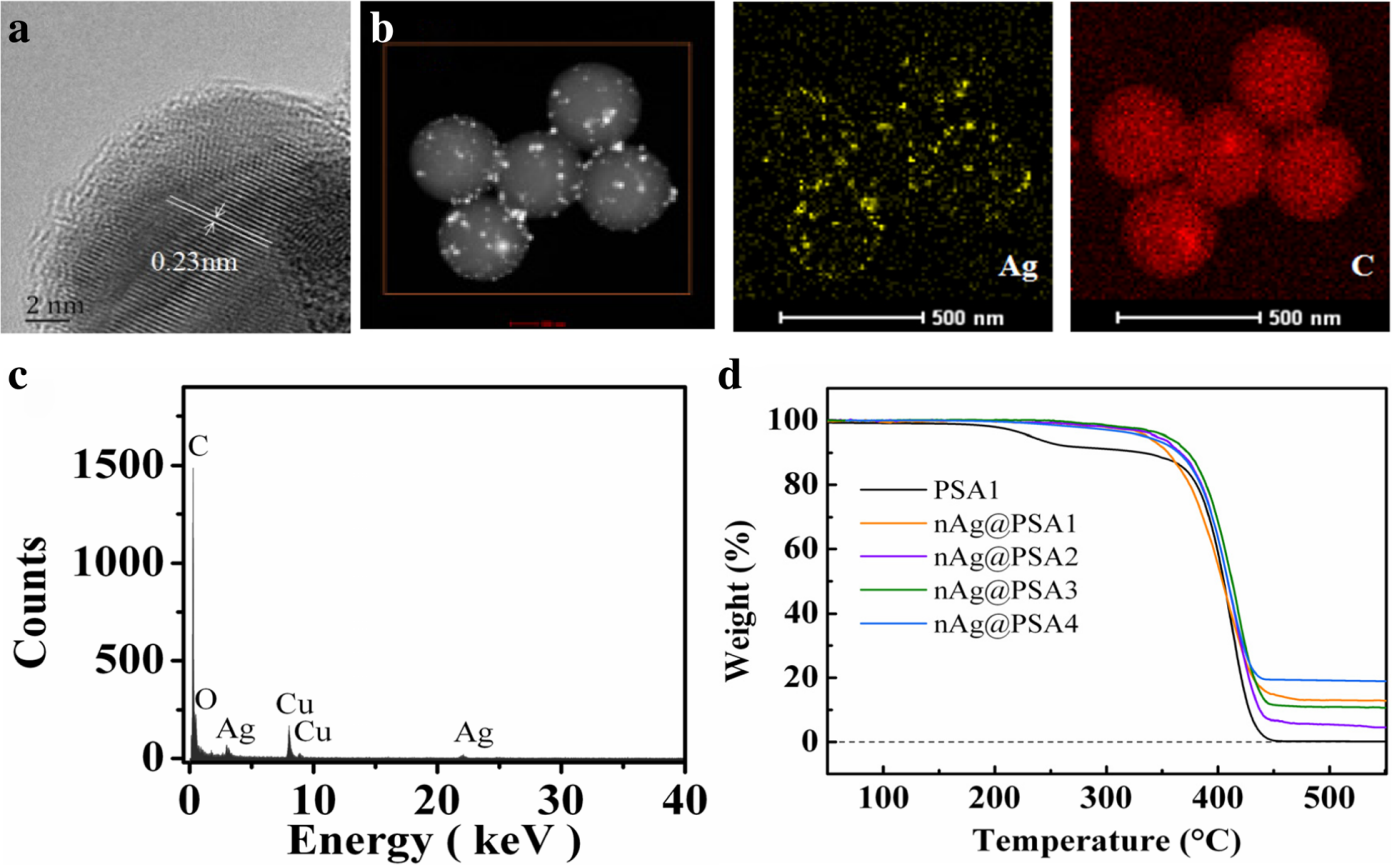
**a** HRTEM image of Ag on PSA4 nanospheres. **b** EDX mapping of nAg@PSA4 composite nanospheres. **c** EDX spectrum of nAg@PSA4 composite nanospheres. **d** TG curves of nAg@PSA1-nAg@PSA composite spheres

PSA1–PSA4 was chosen as basal spheres to prepare nAg@PSA1–nAg@PSA4 composite nanospheres while keeping all other parameters constant (part “Materials and Methods”). TEM micrographs of nAg@PSA1–nAg@PSA4 nanocomposites are shown in Fig. 6. From the micrographs, PSA covered with silver nanoparticles were obvious. As the PSA nanospheres became larger, an increasing number of silver nanoparticles became anchored onto the PSA nanospheres. The coverage and uniformity of the silver nanoparticles on the PSA nanospheres increased accordingly. Figure 7a exhibits the microstructure of Ag nanoparticles covered on PSA nanospheres. The lattice spacing of the silver nanoparticle is 0.23 nm, which agrees with the (111) plane of the silver crystal. This confirmed that Ag nanoparticles were successfully deposited. The deposition of Ag nanoparticles can also be verified from the energy-dispersive X-ray spectroscopy (EDX) data. EDX mapping of the respective Ag and C elements given in Fig. 7b showed the homogenous distribution of these elements. The EDX spectrum confirmed the presence of Ag, C, and O (Fig. 7c). The Cu is from the supporting copper grid.

**Fig. 8:**
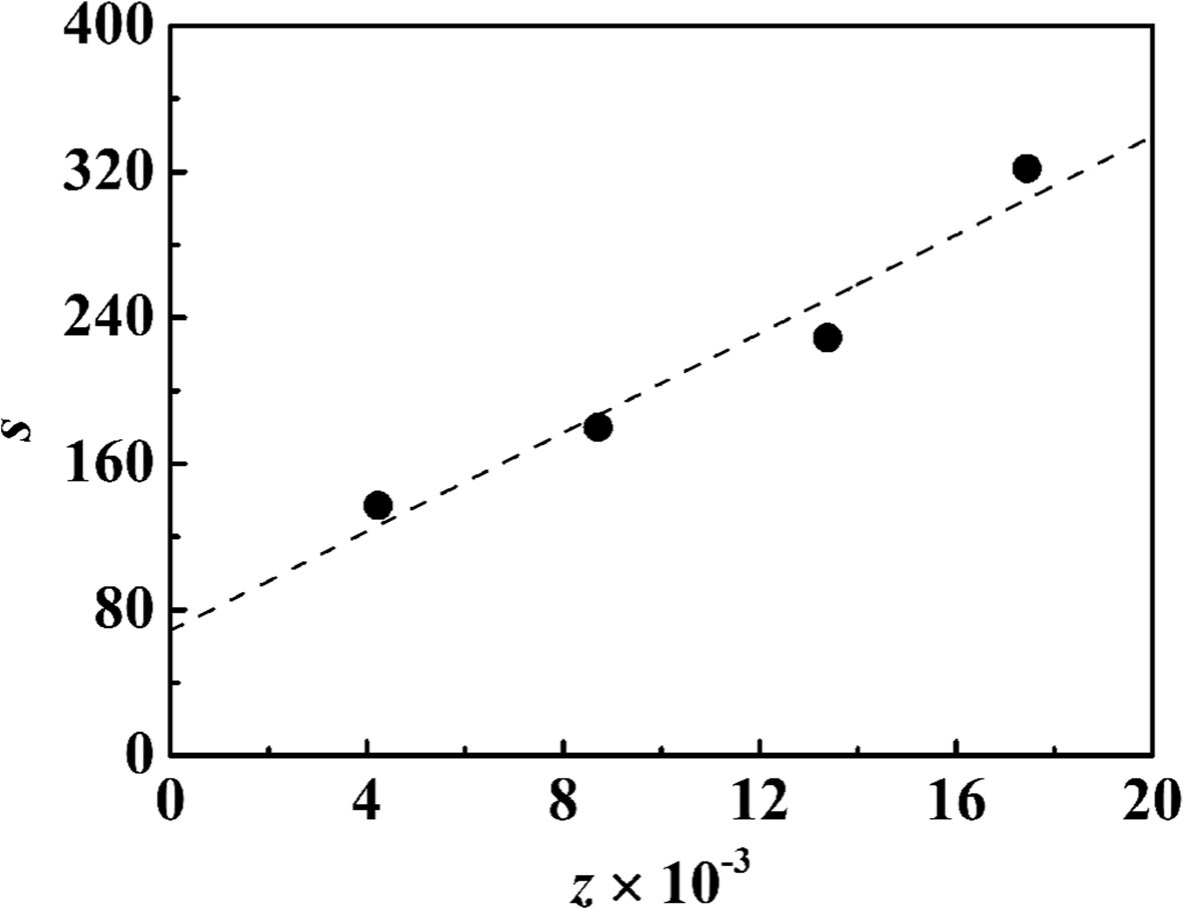
Variation of the amount of silver nanoparticles with number of −COO^*+*^ per particle

Thermogravimetric analysis was employed for further quantitative characterization of silver nanoparticles covered on the PSA nanospheres. TG curves of PSA1 nanospheres and nAg@PSA composite nanospheres obtained from PSA1–PSA4 nanospheres are shown in Fig. 6d. The TG data allowed us to calculate the silver nanoparticles’ weight contribution to the composite nanospheres. We could then estimate the number of silver nanoparticles via the weight *p*. The relationship between the distribution of silver nanoparticles and the dissociation ratio of carboxyl groups is shown in Fig. 8. It is obvious that the average number of silver particles per PSA particle increased linearly with the number of dissociated carboxyl groups. This result may relate to the formation of silver nanoparticles on the surface of composite. The negatively charged carboxyl groups attract the positively charged silver cations via ion pair formation. The attached silver ions are reduced by sodium borohydride, and this is a rapid reducing agent that can induce formation of silver nuclei. The resulting silver nuclei act as nucleation centers; growth occurs by diffusion of silver ions in the solution towards the particle surfaces. These can be interpreted as heterogeneous nucleation/growth sites eventually forming silver nanoparticles. The formation of silver nanoparticles is strongly governed by the balance of the nucleation rate and particle growth.

### Catalytic Performance

**Fig. 9:**
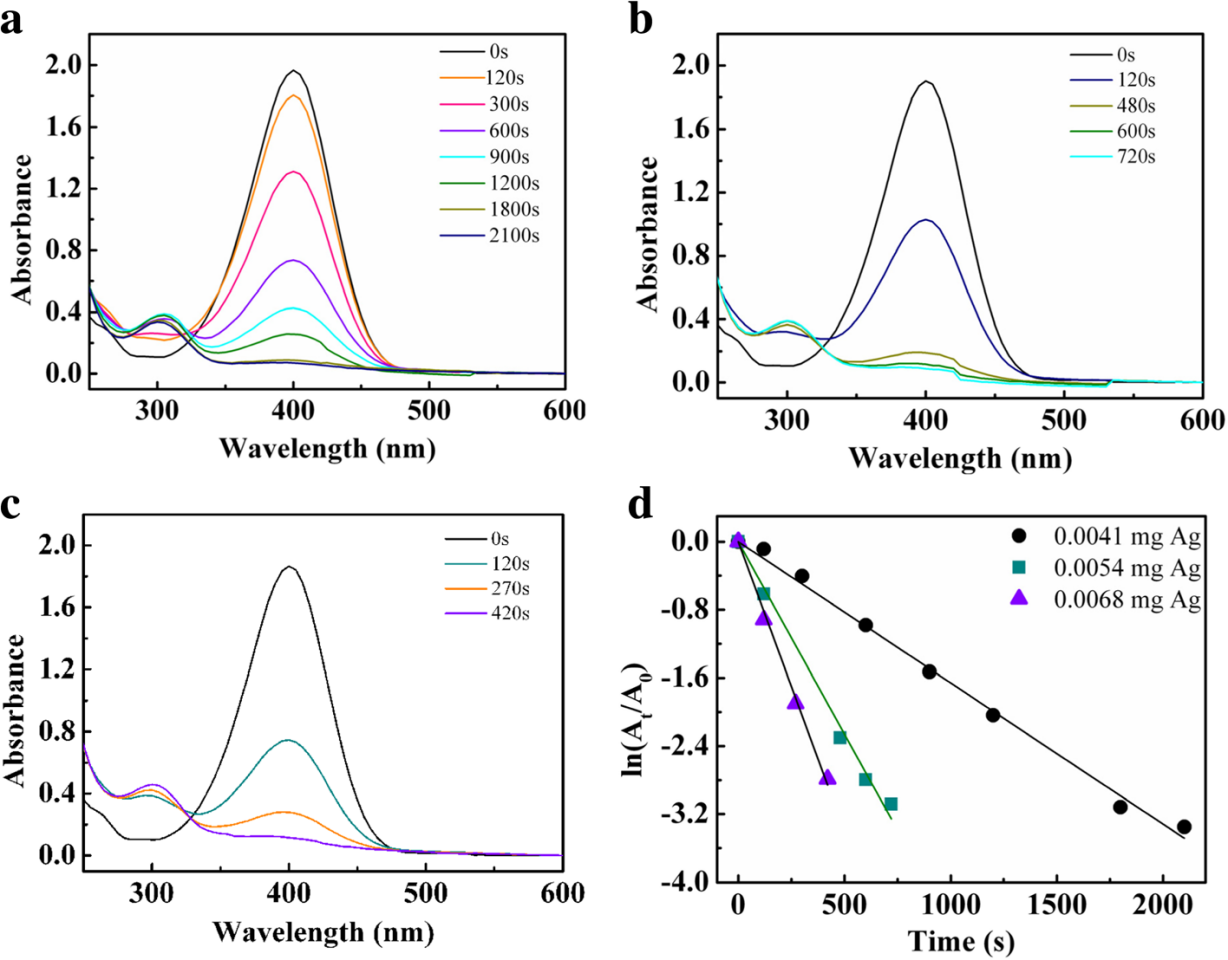
**a** UV-vis spectra of 0.12 mM 4-NP with 36 mM NaBH_4_ in the presence of nAg@PSA4 nanocomposites (containing 0.0041 mg Ag). **b** UV-vis spectra of 0.12 mM 4-NP with 36 mM NaBH_4_ in the presence of nAg@PSA4 nanocomposites (containing 0.0054 mg Ag). **c** UV-vis spectra of 0.12 mM 4-NP with 36 mM NaBH_4_ in the presence of nAg@PSA4 nanocomposites (containing 0.0068 mg Ag). **d** The plot of ln(A_t_/A_0_) against the reaction time in the presence of nAg@PSA4 as a catalyst

The reduction of 4-nitrophenol to 4-aminophenol is a model catalytic reaction and was employed to evaluate the catalytic activity of nAg@PSA nanocomposites. The catalytic reaction was monitored by UV-Vis spectroscopy. The mixture of NaBH_4_ and 4-nitrophenol showed an absorption band at 400 nm corresponding to the 4-nitrophenolate ion. Figure 9a–c illustrated the reduction reaction of 4-nitrophenol observed at different times using different nAg@PSA4 amounts as the catalyst. The intensity of the absorption band at 400 nm gradually decreased and eventually disappeared over time. This is accompanied by the appearance of a new band around 300 nm corresponding to 4-aminophenol. These indicated the conversion of 4-nitrophenol to 4-aminophenol. During this reaction process, the overall concentrations of NaBH_4_ and 4-nitrophenol were 36 mM and 0.12 mM, respectively. The concentration of 4-nitrophenol is proportional to its absorbance; the concentration at reaction time *t* (*C*_t_) and time *t* = 0 (*C*_0_) are equivalent to the absorbance at reaction time *t* (*A*_t_) and time *t* = 0 (*A*_0_). Figure 9d plots ln(*A*_t_/*A*_0_) versus reaction time in seconds. The results indicated that ln(*A*_t_/*A*_0_) decreased linearly with time. This follows a pseudo-first-order kinetic behavior. The rate constant *k* at room temperature was calculated from the slope, and the constants of nAg@PSA4 containing 0.0041 mg Ag, nAg@PSA4 containing 0.0054 mg Ag, and nAg@PSA4 containing 0.0068 mg Ag were 1.66 × 10^− 3^ s^−1^, 4.52 × 10^−3^ s^−1^, and 6.80 × 10^−3^ s^−1^, respectively. These results showed that the more the catalyst amount, the faster the reaction rate. The largest rate constant *k* at room temperature is comparable to the counterpart of previously reported Ag nanocomposite catalysts, such as 0.7 × 10^−2^ s^−1^ of G_4_-PAMAM-NH_2_(Ag_12_) [28], 1.274 × 10^−2^ s^−1^ of Ag_10_@SBA-15 [29], 6.2 × 10^−3^ s^−1^ of CNFs/AgNPs [30], and 31.64 × 10^−2^ min^−1^ of [AgCl_2_]^−^ complex [31].

**Fig. 10:**
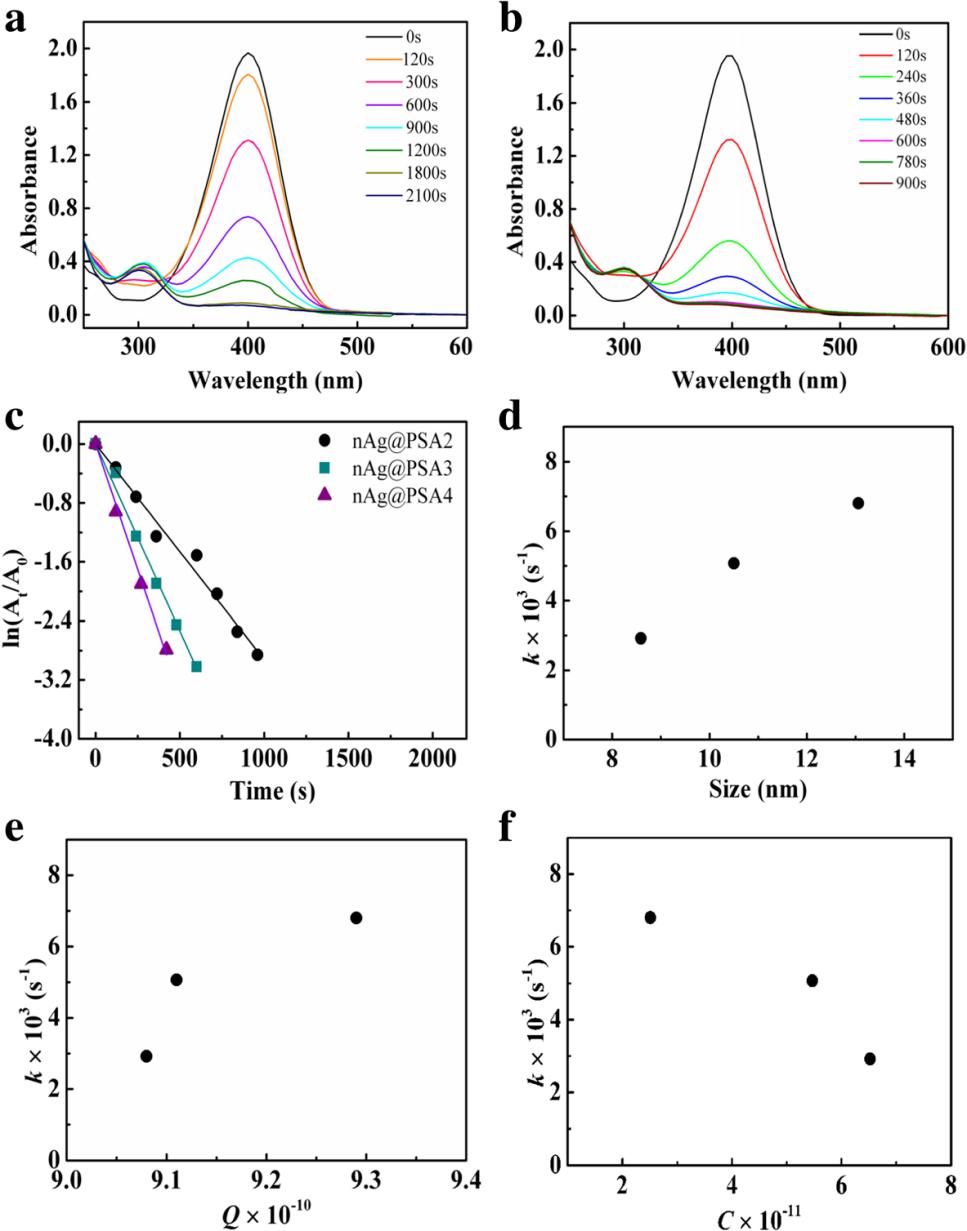
**a** UV-vis spectra of 0.12 mM 4-NP with 36 mM NaBH_4_ in the presence of nAg@PSA2 nanocomposites (containing 0.0068 mg Ag). **b** UV-vis spectra of 0.12 mM 4-NP with 36 mM NaBH_4_ in the presence of nAg@PSA3 nanocomposites (containing 0.0068 mg Ag). **c** Plot of ln(A_t_/A_0_) against the reaction time in the presence of nAg@PSA nanocomposites (containing 0.0068 mg Ag) obtained from PSA2–PSA4 nanospheres. **d** Effects of the particle size of silver nanoparticles on the rate constant. **e** Effects of the silver amount per square centimeter of PSA nanospheres surface on the rate constant. **f** Effects of the nanosphere amount per cubic centimeter of nAg@PSA suspension on the rate constant

The reduction reactions of 4-nitrophenol, using nAg@PSA2 and nAg@PSA3 as the catalyst, are shown in Fig. 10a, b. With the same addition of silver amount, the rate constants of nAg@PSA2, nAg@PSA3, and nAg@PSA4 were 2.92 × 10^3^ s^−1^, 5.07 × 10^3^ s^−1^, and 6.80 × 10^3^ s^−1^, respectively (Fig. 10c). These results demonstrate that the catalytic performance of nAg@PSA increases with the increase of diameter of PSA nanospheres, or the number of silver particles per PSA particle. With the increase in the diameter of PSA sphere, the as fabricated catalytic film would be more polyporous with the higher dispersity of Ag nanoparticles. Higher dispersity offers more opportunity for reactants reaching to surface of Ag nanoparticles. Figure 10d, e shows that the reaction rates increased with increasing silver particle size and amount of silver per square centimeter of PSA nanospheres surface, respectively. Figure 10f showed that the reaction speed increases with smaller amounts of nanospheres per cubic centimeter of nAg@PSA suspension.

**Fig. 11:**
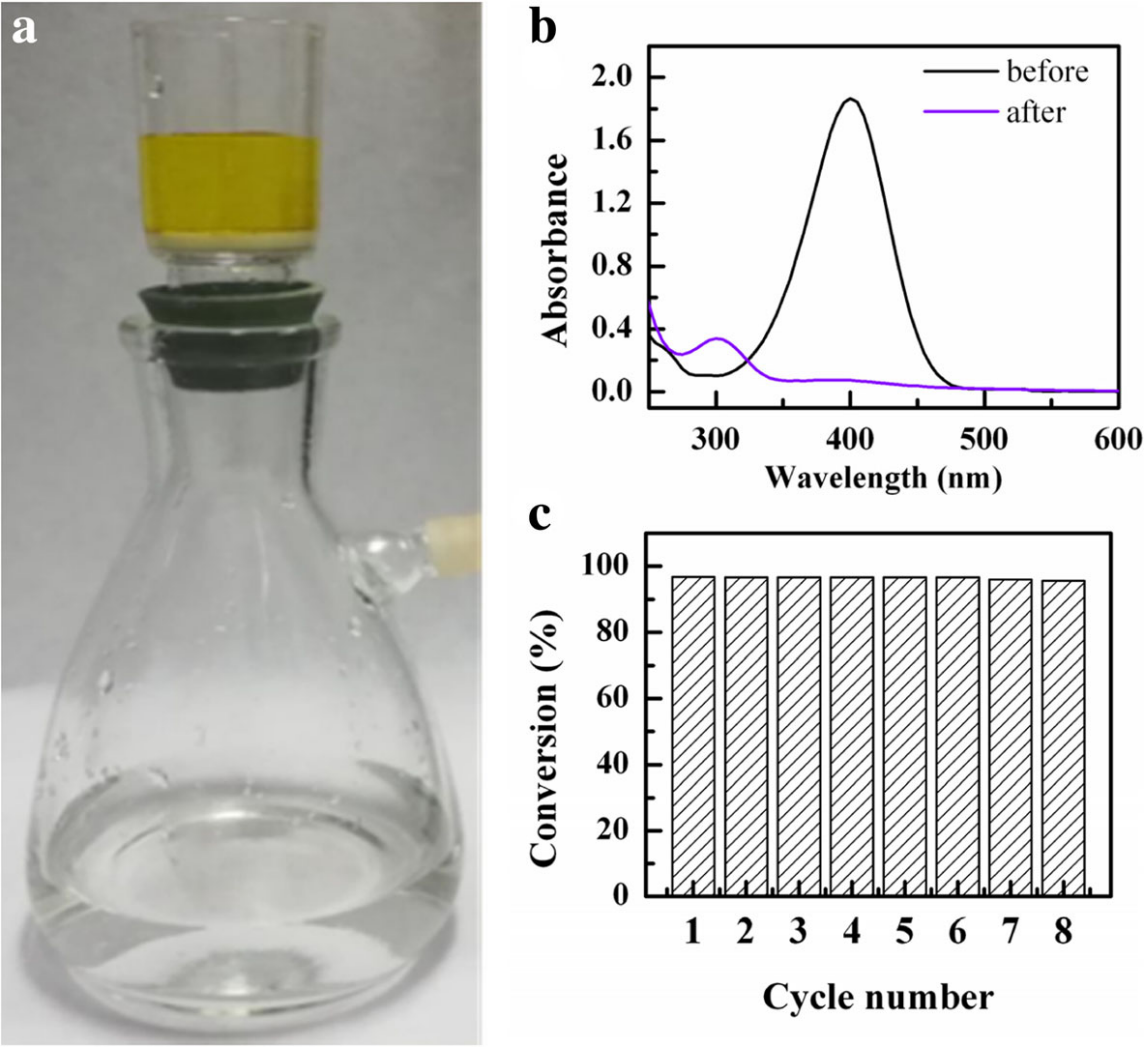
**a** Photograph showing the fast reduction of 4-NP passing through an nAg@PSA catalytic membrane. **b** UV-vis spectra of 0.12 mM 4-NP with 36 mM NaBH_4_ before and after passing through the nAg@PSA catalytic membrane. **c** The conversion rate of 4-NP during the cyclic testing

To evaluate the reusability, nAg@PSA nanocomposites were deposited on a glass sand funnel to form a catalytic membrane. When the reaction mixture (36 mM NaBH_4_ and 0.12 mM 4-NP) passed through the membrane, the yellow color faded in the reaction mixture demonstrating a fast catalytic reaction (Fig. 11a). The conversion rate of 4-nitrophenol was determined by *A*_t_/*A*_0_ at *λ* = 400 nm. According to Fig. 11b, the conversion rate was 96% after passing through the catalytic membrane. Moreover, from Fig. 11c, the catalytic membrane was active up to eight cycles of 4-nitrophenol reduction—this confirms the high reusability of the catalyst.

## Conclusions

We synthesized monodisperse poly(styrene-*co*-acrylic acid) (PSA) nanospheres via soap-free emulsion polymerization, and the nAg@PSA composite nanospheres could be facilely prepared through the in situ reduction of silver nitrate via sodium borohydride in aqueous solution. SEM micrographs of PSA nanospheres indicated that the PSA nanospheres were spherical with a narrow particle size distribution. The plot of carboxyl against the diameters was linear with a slope of 2.0. This indicates that the carboxyl groups are mainly distributed on the surface of PSA nanospheres. Dissociation should occur on the particle surface rather than within the particle volumetrically because the amount of dissociated carboxyl groups is proportional to the surface area of the latex particle. The amount (*z*) of dissociated carboxyl groups is 1% of the amount (*n*) of carboxyl groups. TEM confirmed the formation of silver nanoparticles coated onto the PSA nanospheres. The dissociated charges on the surfaces of PSA nanospheres had a major influence on the coverage of Ag nanoparticles on PSA nanospheres. The catalytic performance of nAg@PSA nanospheres was investigated in catalyzing the reduction of 4-nitrophenol. These synthesized nAg@PSA nanospheres contained highly dispersed silver nanoparticles with high catalytic activity and good recyclability.

## Materials and Methods

### Materials

Acrylic acid (AA), potassium persulfate (KPS), sodium borohydride (NaBH_4_), silver nitrate (AgNO_3_), and sodium hydroxide were purchased from Aladdin Chemical Reagent Co., Ltd. (Shanghai, China). Styrene (St) was supplied by Tokyo Chemical Industry Co., Ltd. (Tokyo, Japan). All chemicals were of analytical grade and were used as received unless specified.

### Synthesis of PSA Nanospheres

PSA nanospheres were prepared according to the literature [22–24, 32, 33]. Typically, AA and H_2_O (130 mL) were initially charged into a glass flask. Styrene was added after the dissolution of feeded AA. The flask was then heated to 75 °C with stirring under nitrogen condition. Polymerization was initiated after KPS solution (20 mL) was injected and then maintained at 75 °C for 12 h. Other operation parameters were shown in Table 2. The products were purified by seven cycles of centrifugation–redispersion in distilled water and then finally dispersed in water.

**Table 2:** Recipes of PSA nanospheres

Sample code	AA (g)	St (g)	KPS (g)	Stirrer speed (r min^−1^)
PSA1	0.9	6	0.12	400
PSA2	0.3	6	0.48	300
PSA3	0.3	6	0.24	300
PSA4	0.9	18	0.24	300

### Preparation of nAg@PSA Composite Nanospheres

The typical procedure for fabricating nAg@PSA nanocomposites [22–24] is described as follows: PSA dispersion (500 mL, 0.3 mg ml^−1^) was mixed with aqueous solution of AgNO_3_ (12.5 mL, 10 mM) in the glass flask. The mixture dispersion was stirred at 300 r min^−1^ for 5 h at room temperature. After that, NaBH_4_ (12.5 mL, 10 mM) was added to the dispersion and the resulting mixture was allowed to react at 0 °C for 2 h with stirring. The suspension was centrifuged (20 min, 12,000 r min^−1^), and the precipitate was washed with distilled water (30 mL). This centrifugation–redispersion cycle was repeated four times to remove impurities.

### Catalytic Performance Experiments

The reaction of 4-nitrophenol by sodium borohydride catalyzed with nAg@PSA nanocomposites was performed in aqueous solution. The reaction procedure was as follows: NaBH_4_ aqueous solution (4.5 mL, 80 mM) was mixed with 4-NP solution (0.5 mL, 2.4 mM) in a 10-mL Eppendorf tube. Then, the nAg@PSA nanocomposites were added into the mixture solution, and the volume was adjusted to 10 mL with distilled water. Immediately after, an aliquot was placed in a 1-cm path length quartz cell for UV-vis spectroscopy.

The nAg@PSA4 suspension (containing 1.3538 mg Ag) was filtered through a glass sand funnel equipped with filter conical flask, deposited on a filter paper, and washed with distilled water (100 mL). The 10 mL of solution (36 mM NaBH_4_ and 0.12 mM 4-NP) was subjected to vacuum filtration in the funnel covered by the nAg@PSA4 membrane. The collected solution in the conical flask was characterized with UV-Vis spectroscopy. To evaluate the reusability of the nAg@PSA4 catalyst, the membrane was washed with distilled water and reused.

### Distance-Variable Parallel Electrodes System and Instruments

**Fig. 12:**
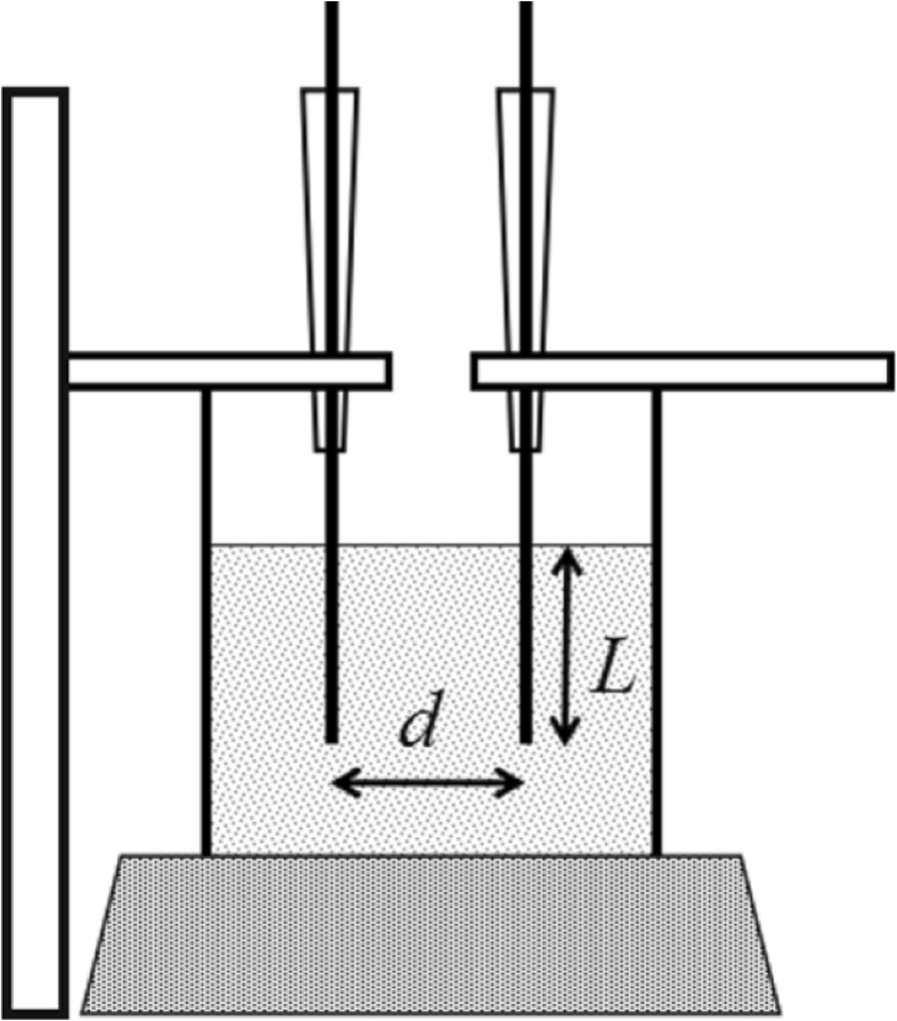
Illustration of the parallel distance-variable electrode system

Distance-variable parallel electrodes systems were composed by two platinum wires 0.1 mm in diameter and an xz positioner. As illustrated in Fig. 12, one of the wires was fixed, and the distance *d* was adjusted by moving the *y* axis of the xz positioner. The length is immersed in solution controlled by the *z* axis of the xz positioner. AC impedance with a different distance was obtained, according to our previous works [25–27].

The nanoparticle features and the location of the silver particles on the latex surface were investigated by SEM and TEM (JEOL JEM-2010, JEOL Ltd., Tokyo, Japan). The content of silver nanoparticle coatings on PSA nanospheres was analyzed by thermogravimetric analysis (Perkin Elmer Pyris 1, Perkin-Elmer Co., USA). The UV-Vis spectra were recorded on Shimadzu UV-2550 spectrophotometer (Shimadzu, Kyoto, Japan) at room temperature.

## Abbreviations

*a*: The radius of Pt wire
*d*: The distance between two electrodes
*D*_H_ and *D*_L_: The diffusion coefficients of hydrogen ion and −COO^—^ carrier PSA sphere, respectively
*L*: The length of Pt wire immersed in suspension
*n*: The number of −COOH per PSA microsphere
nAg@PSA: Silver nanoparticle-loaded poly(styrene-co-acrylic acid) nanocomposites
PSA: Poly(styrene-co-acrylic acid) nanospheres
*z*: The number of –COO^—^ per PSA microsphere
*Λ*: The molar conductivity of the PSA suspension with concentration *c*
